# Implementing the ACHIEVE Model to Prevent and Reduce Chronic Disease in Rural Klickitat County, Washington

**DOI:** 10.5888/pcd10.120272

**Published:** 2013-04-18

**Authors:** Laura Horne, Katie Miller, Sandra Silva, Lori Anderson

**Affiliations:** Author Affiliations: Katie Miller, Public Health Institute, Oakland, California; Sandra Silva, Altarum Institute, Washington, DC; Lori Anderson, Klickitat County Planning Department, Goldendale, Washington.

## Abstract

**Background:**

In the United States, 133 million people live with 1 or more chronic diseases, which contribute to 7 of 10 deaths annually. To prevent and reduce the prevalence of chronic diseases, the National Association of County and City Health Officials (NACCHO) provided technical assistance and funding to 33 local health departments in Washington State, including the Klickitat County Health Department (KCHD), to implement the Action Communities for Health, Innovation, and Environmental Change (ACHIEVE) model.

**Community Context:**

Klickitat County residents experience higher rates of obesity and overweight than people living in urban areas in the state. KCHD applied the ACHIEVE model to accomplish 2 objectives: 1) to engage the community in community health assessment, action plan development for chronic disease prevention, and implementation of the plan and 2) to work with targeted sectors to promote worksite wellness and to establish community gardens and bicycling and walking trails.

**Methods:**

KCHD convened and spearheaded the Healthy People Alliance (HPA) to complete a community assessment, develop a community action plan, implement the plan, and evaluate the plan’s success.

**Outcomes:**

KCHD, working with HPA, accomplished all 5 phases of the ACHIEVE model, expanded a multisector community coalition, developed Little Klickitat River Trail and 3 community gardens, and created and promoted a worksite wellness toolkit.

**Interpretation:**

Assistance and training that NACCHO provided through ACHIEVE helped the KCHD engage nontraditional community partners and establish and sustain a community coalition.

## Background

In the United States, 133 million people live with 1 or more chronic diseases, which contribute to 7 of 10 deaths annually (1). Chronic diseases result in $1 trillion in US health care expenditures, and that number is expected to rise to $4.2 trillion over the next 15 years (2,3). Obesity, which can result from poor nutrition and low physical activity, is a measure and predictor of chronic diseases such as heart disease and diabetes (4).

Rural Americans are disproportionately affected by overweight and obesity. Children in rural areas are 25% more likely to be overweight or obese than children in urban areas, and rural Americans are also more likely to live in poverty, have sedentary lifestyles, and lack access to health insurance and preventive health care (5). These factors, in addition to high cost or limited availability of healthful foods and access to recreational activities, contribute to higher rates of obesity among rural populations.

With support from the Centers for Disease Control and Prevention (CDC), the National Association of County and City Health Officials (NACCHO) provided technical assistance and funding to 33 local health departments, including the Klickitat County Health Department (KCHD) in Washington State, to improve community health through a collaboration called Action Communities for Health, Innovation, and Environmental Change (ACHIEVE).

ACHIEVE addresses chronic disease risk factors, specifically physical activity, nutrition, and tobacco use, through policy, systems, and environmental approaches. Implementation requires collaboration among multiple sectors, including worksites, health care organizations, schools, and community institutions. The ability to create or strengthen coalitions of community partners across public and private sectors is key to success. ACHIEVE’s model for community engagement (Figure) guides communities through health improvement.

**Figure:**
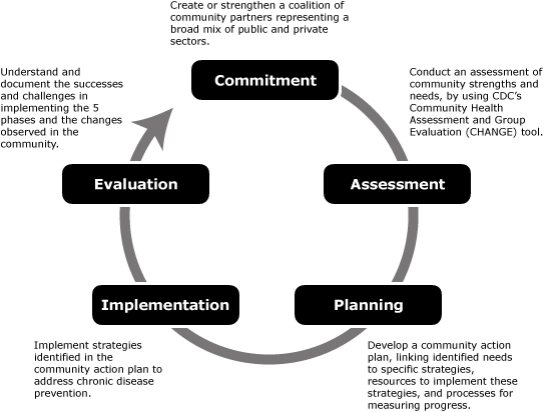
The 5 phases of Action Communities for Health, Innovation, and Environmental Change (ACHIEVE), developed by the Centers for Disease Control and Prevention.

## Community Context

Klickitat County, 1 of 39 counties in Washington State, comprises 1,904 square miles. Half of its estimated population of 20,697 reside in 3 cities, Bingen, Goldendale, and White Salmon; the other half reside in unincorporated areas. Since the decline of the timber industry in the 1980s, the unemployment rate in Klickitat County has been higher than that of most counties in the state. In June 2012, the unemployment rate in Klickitat County was 10.8%, and the state rate was 9.6% (6). Nearly 1 in 5 Klickitat County residents lives below the federal poverty level and earns less than $14,580 annually for a family of 2 and less than $22,056 for a family of 4 (7). Whites make up 93% of the Klickitat County population; Hispanics, the largest ethnic minority, make up 8%; and American Indians, 2.7%. Asians, blacks, and a small number of other racial/ethnic minorities make up the remainder (7).

Klickitat ranks 22nd of 39 Washington State counties in overall health. Residents experience higher rates of obesity and overweight than people in more urban areas within the state. County Health Rankings (6) reports that 27% of Klickitat adults are obese (body mass index [BMI] ≥30 kg/m^2^), and 23% report no leisure time physical activity (6); they also tend to be older, less educated, and have lower incomes than urban residents, all factors related to higher obesity rates.

High food costs and limited safe opportunities for physical activity restrict healthful lifestyles and contribute to obesity. Opportunities for low-income families to purchase affordable, healthful food are scarce in Klickitat County. Fast-food establishments make up 26% of restaurants; a community food assessment conducted from 2007 to 2009 found that 27% of residents travel more than 26 miles to shop for affordable, diverse groceries. Other options for affordable nutrition include participation in community gardens in surrounding counties or a food pantry. Safe, affordable opportunities to be physically active are also difficult to find. A 2007 community environment assessment conducted by the Washington State in several Klickitat County towns showed few places to walk or bicycle and few community groups supportive of low-cost physical activities (8). In 2002, 6.7% of 10th grade students in Klickitat County were overweight; this rate doubled in 2 years among the same group of students (9).

NACCHO provided funding, training, and assistance to KCHD to accomplish 2 objectives: to engage the community in a process of assessment, planning, and implementation and to work with targeted sectors to establish community gardens and bicycling and walking trails, and to promote worksite wellness. These objectives conform to the ACHIEVE model, which calls for commitment (establish a coalition and develop a common vision), assessment (complete a health assessment of local schools, worksites, health care facilities, community institutions, and the community-at-large), planning (review assessment results, prioritize goals, and develop a community action plan), implementation (implement the community action plan), and evaluation (assess implementation of the community action plan).

## Methods

### Phase 1: Commitment

In 2008 NACCHO selected KCHD to participate in the 3-year ACHIEVE model. KCHD first attended coalition leadership training and developed a coalition of community leaders across sectors. KCHD invited partners to participate in the coalition and initially secured commitment from 11 public health, government, and health care representatives. In July 2009, these 11 members attended a follow-up training led by CDC, NACCHO, and other national partners to learn the ACHIEVE model.

Coalition members developed a structure and process to involve agencies and community members in decisions. Members decided on a common vision, mission, and new name, Healthy People Alliance (HPA). Members represented both ends of the county, the “modern and hip” western part of the county and the more rural and traditional central and eastern parts of the county. In light of the physical distance and philosophical differences from one end of the county to the other, HPA developed 2 subgroups for the central/east and west sides of the county. Each subgroup elected a chairperson, co-chair, and secretary and met monthly. Officers from both subgroups constituted the executive board and met quarterly to discuss coalition-wide activities. Prior to HPA’s establishment, there had been few coalition efforts to address chronic disease in Klickitat County.

### Phase 2: Assessment

After training, HPA used CDC’s Community Health Assessment and Group Evaluation (CHANGE) tool (10) to conduct an assessment of health assets and needs in schools, worksites, health care facilities, community institutions, and the overall community (Table 1). Coalition members collected and analyzed data from interviews, observations, and surveys in each sector and supplemented CHANGE with data from the 2010 US Census (7), 2002 Washington State Healthy Youth Survey (9), and local health data available through KCHD and health care organizations. HPA reported its members remained passionate and engaged in the assessment while handling their full-time jobs and other community work.

**Table 1:** Community Assets and Needs Identified with the CHANGE Tool, by Sector, Healthy People Alliance, Klickitat County, Washington, 2009–2011

Sector	Assets	Needs
Schools	Comprehensive tobacco-use policies; staff training to assist students with chronic disease self-management; strong social network; space for gardens	Support in developing, implementing, and funding healthful eating and physical activity strategies
Worksites	Strong adherence to community-wide tobacco-use policies; interest in supporting employee wellness	Leadership in health promotion and chronic disease prevention; worksite wellness programs
Community-at-large (Klickitat County)	Strong tobacco-use policies; locally grown healthful foods; spaces for physical activity	Funds; time and transportation to access existing nutrition and physical activity opportunities; infrastructure to support walking and biking
Health care facilities	Interest in promoting wellness within the facilities and in the community	Worksite wellness programs
Community institutions or organizations	Leadership in health promotion; strong adherence to community-wide tobacco-use policies; strong network of community members	Some have policies and environmental structures supporting healthful eating and physical activity and others do not

Abbreviation: CHANGE, Community Health Assessment and Group Evaluation.

### Phase 3: Planning

HPA identified and discussed key assets and needs within each sector. Although the county possessed local, healthful foods and spaces for physical activity, cost and lack of time and transportation limited access to these resources. The county also lacked infrastructure to support safe walking and bicycling. Additionally, although business leadership was not strongly engaged in chronic disease prevention and management, employers seemed interested in worksite wellness. HPA identified 2 worksites, Klickitat County and Klickitat Valley Health (KVH) that were in the process of developing worksite wellness programs. Both entities requested support from HPA in evaluating and enhancing their wellness programs.

HPA identified strategies to increase access to healthful foods and places to engage in physical activity on the basis of needs identified in the community assessment, membership interest, and activities already under way. The coalition included 3 prioritized strategies in the action plan: to increase coalition membership with diverse cross-sector representation, to establish community gardens and enhance a biking and walking trail, and to develop and promote a local worksite wellness toolkit. HPA also identified evaluation measures and data sources for each strategy (Table 2). For example, to assess success of community gardens and the trail, the coalition planned to conduct focus groups and take an inventory of county-wide parks and recreation opportunities.

**Table 2:** Data Sources and Outcomes for Priority Strategies, Healthy People Alliance, Klickitat County, Washington, 2009–2011

Priority Strategy	Sector Affected	Data Sources for Measuring Progress	Outcomes	Future Plans
Increase coalition membership with diverse cross-sector representation	Community-at-large	Coalition membership list and tracking sheet for number of community events and film screenings	Coalition membership grew from 11 to 35 members. Members were recruited through 4 film screenings and 18 community events. Potential number of people reached: 500 people attended community events.	To focus on reaching more of the rural populations in outlying areas, develop more resources for children and youth, and broaden website and social media campaign.
Establish community gardens	Community institutions/organizations	Implementation of garden and a focus group with community garden participants	HPA partnered with Head Start (Goldendale and White Salmon) and the Guided Path Transitional Housing (White Salmon) to develop community gardens. Potential number of people reached: 60 garden participants.	HPA provides ongoing technical assistance and support to the participating organizations. The gardens continue to expand, and HPA will pursue a partnership with a local food pantry.
Enhance a biking and walking trail	Community-at-large	Inventory of county-wide parks and recreation opportunities, number of new active living partnerships developed, number of trails enhanced	HPA developed new partnerships with the parks and recreation district and the State Department of Fish and Wildlife. HPA completed a recreational needs assessment and enhanced Little Klickitat River Trail. Potential number of people reached: 4,000 residents in the district where the trail is located.	HPA will continue their work to promote active living through Complete Streets and Safe Routes to School initiatives, made possible through an Active Community Environment Grant from the state departments of transportation and health.
Develop and promote a local worksite wellness toolkit	Worksites	Development of worksite wellness toolkit, tracking sheet for number of worksites identified who are interested in implementing a wellness program, presentation of the toolkit to those worksites	A volunteer and HPA’s Healthy Worksite Committee completed the Worksite Wellness Toolkit for Klickitat County. Washington State Department of Health launched the toolkit at a worksite symposium in Goldendale and White Salmon. Potential number of people reached: 45 worksite representatives in attendance at symposium.	HPA will provide ongoing technical assistance to worksites that would like to implement the toolkit

Abbreviation: HPA, Healthy People Alliance.

### Phase 4: Implementation

Recruitment in unincorporated areas was a challenge. Increasing awareness through community events, the local farmers market, and town council meetings helped address this challenge. HPA partnered with community organizations to host 4 screenings of *Unnatural Causes* (11), a documentary exploring racial and socioeconomic inequalities in health. HPA members also attended 18 community events where they provided membership and e-mail newsletter sign-up forms with appropriate marketing materials based on the topic of the community event. To inform the public, HPA launched a website (www.healthypeoplealliance.org) and a Facebook marketing campaign. HPA also provided a summary of its action plan during community events and scheduled meetings with county commissioners to update them on the coalition’s work. County commissioners were skeptical at first because of tough economic times, but they grew supportive of HPA because of funding offered through NACCHO and dedication of HPA members.

With funds from a National Park Service Planning Grant and the assistance of a National Park Service planner who lived in the county, HPA conducted a recreational needs assessment in the Goldendale Parks and Recreation District in the fall of 2010. HPA discovered an opportunity to further develop Little Klickitat River Trail to provide community members a safe and accessible walking trail. HPA purchased educational signage, funded the construction of gravel paths and bridges to make the trail accessible for people with disabilities, and began construction. Signage included a trail map, plant identification signs, and information on the area’s history, fish, and wildlife.

HPA sought to reach children and low-income residents in Klickitat County and increase their knowledge of and access to fresh fruits and vegetables. This led to partnerships with the Head Start program in Goldendale and White Salmon and with the Guided Path Emergency Shelter and Transitional Housing program in White Salmon to develop community gardens in the spring of 2011. HPA requested a wish list from these programs, purchased compost and seeds, and provided assistance to the garden sites. Head Start staff incorporated the gardens into their curricula and obtained seed donations from other organizations. At the transitional housing facility, coalition members mentored residents and staff on gardening and referred participants to other local community gardens to ensure residents could continue after leaving transitional housing. Residents also had the opportunity for container gardening, which was transferable to any home.

Simultaneous with development of community gardens, a KCHD volunteer worked with HPA’s Healthy Worksite Committee, which was composed of central/east and west subgroup members interested in collaboration on healthy worksites, to develop a worksite wellness toolkit on physical activity and nutrition. The volunteer and a representative from the Washington State Health Department conducted a presentation on healthy worksites to 45 local business representatives, including Klickitat County, Goldendale School District, and Klickitat Valley Health, to launch the toolkit at symposiums in Goldendale and White Salmon. HPA also posted the toolkit on the HPA website and presented it to worksites identified as interested in wellness programs through the CHANGE assessment. HPA worked with the county’s 2 chambers of commerce in Goldendale and Mount Adams and with a local business network to promote the toolkit. Long-term plans include incorporation of strategies for healthful living into business models and recognition of healthy businesses through a certification program.

## Outcome

NACCHO contracted with Altarum Institute in Washington, DC, to conduct process-level evaluation of 33 ACHIEVE coalitions, including HPA. Altarum administered annual Web-based surveys and telephone interviews each fall during the funding period (2009–2011) to document progress of several outcomes identified as precursors to behavioral changes leading to reduced chronic disease rates. One representative from each funded local health department (33 since 2009) was invited to participate in an online survey and telephone interview; all invited representatives participated in both evaluation methods. Given the short funding period, evaluation focused on short and intermediate outcomes, such as changes in collaboration, community engagement, messaging, awareness, policy, systems, and environmental strategies in the community. For HPA, evaluation focused on their 3 priority strategies.

Surveys completed by coalition leaders showed an improvement in coalition structures over the 3-year period, including vision and mission statements, goals, policies, member roles, a planning structure, and communication mechanisms. The west and central/east subgroup structure has worked well for HPA, and HPA has obtained Internal Revenue Service 501(c)3 status.

HPA reported strengthened leadership, knowledge, and skills among coalition members as the organization participated in trainings, received assistance, and gained experience during implementation of priority strategies. By 2011, HPA expanded to include nonprofit organizations, faith-based organizations, businesses, and conservation and environmental groups (Table 3).

**Table 3:** Coalition Membership List, Healthy People Alliance, Klickitat County, Washington, 2009–2011

Organization Name	Organization Role	Organization Type	Sector	Project Year Joined
Mid-Columbia Children’s Council; Klickitat Trail Conservancy	Supervisor; president	Nonprofit organization; community-based organization	Work-site; community-at-large	2009–2010
Goldendale Ministerial Association	Pastor	Faith-based organization	Community-at-large	2009–2010
Klickitat County Health Department	Nursing director	Public health organization	Health care	2009–2010
Klickitat Valley Health; private counseling practice	Counselor	Health care organization; other (private practice)	Health care	2009–2010
Klickitat Valley Health	Commissioner	Health care organization	Health Care	2009–2010
Central Klickitat Park and Recreation District	Board member	Government organization	Community-at-large	2009–2010
Klickitat County Planning Department; Central Klickitat Park and Recreation District	Staff member; board member	Government organization	Community-at-large	2009–2010
Klickitat County Health Department	Chronic disease prevention coordinator	Government organization	Community-at-large	2009–2010
Department of Social and Health Services, Goldendale Community Services	Office administrator	Government organization	Community-at-large	2009–2010
Klickitat County Health Department	Public health nurse	Government organization	Community-at-large	2009–2010
Skyline Hospital	Nurse	Health care organization	Health care	2009–2010
Community	Independent contractor	Individual	Community-at-large	2009–2010
Klickitat Valley Health	Radiology department	Health care organization	Health care	2009–2010
Public Utility District	Human resource manager	Business/for profit	Community-at-large	2009–2010
Klickitat Valley Health	Nurse/diabetes educator	Health care organization	Health care	2009–2010
Community	Community member	Individual	Community-at-large	2009–2010
Klickitat County Health Department; AmeriCorps	Community member	Government organization	Community-at-large	2010–2011
Washington State University Horizons	Community member	Nonprofit organization; community-based organization	Community-at-large	2010–2011
Mountain Sage Medicine	Naturopath	Business/for profit	Community-at-large	2010–2011
Mid-Columbia Children’s Council Head Start	Teacher	Nonprofit organization; community-based organization	Community-at-large	2010–2011
Mid-Columbia Children’s Council Head Start	Teacher	Nonprofit organization; community-based organization	Community-at-large	2010–2011
Klickitat County Health Department	Environmental health specialist	Government organization	Community-at-large	2010–2011
Mount Adams Park and Recreation District	Commissioner	Government organization	Community-at-large	2010–2011

Altarum invited all 35 HPA members to complete the Web-based Wilder Collaboration Factors Inventory survey (12), which addresses 20 factors shown to influence coalition success. An average of 5 HPA members completed the survey annually. Members’ scores on a scale of 1 to 5, with 5 being the highest score, were averaged for each factor. Although data suggests HPA improved on all factors for successful collaboration, results should be interpreted with caution given low response rates.

Beyond improvements within the coalition, HPA also reported significant changes in community education about chronic disease, education with key decision makers and stakeholders, engagement of new partners, and social media activities to increase visibility.

Telephone interviews with coalition leaders revealed some of the leaders’ challenges. An ongoing challenge was limited availability of coalition members. As part of a rural community, coalition members were often involved in multiple community efforts with limited ability to participate in all activities. Despite this challenge, coalition leaders advanced activities through a sufficient level of involvement, consistent meetings, and communication. They doubled coalition membership and completed all goals in the community action plan. Since the establishment of the Goldendale community garden, HPA has provided a water faucet and deer-proof fence to double the garden’s size and has started a program to donate extra produce to the local food bank.

The need for additional funding was another challenge. HPA focused on seeking grant opportunities and in 2011 secured an Active Community Environment grant from the Washington State Department of Transportation to implement policies supporting safe walking and bicycling within the community. HPA reported new community partnerships, in addition to strong leadership from coalition chairs, as factors that facilitated success. Assistance and training on coalition-building and partnership development provided through ACHIEVE helped HPA to involve key stakeholders in their work for the first time.

Coalition successes and opportunities for involvement are distributed through the HPA website, social media, and coalition meetings. HPA was surprised by the effectiveness of social media promotions. The coalition initially speculated that rural communities did not use the Internet or social media. However, the positive response they received from the community as a result of social media demonstrated its use.

## Interpretation

Since 2009, HPA has developed into a strong coalition with strengths in decision making, communication, and adaptability. Assistance and training provided through ACHIEVE brought in new partners to adopt its vision for a healthier community. Funding also allowed KCHD to lead coalition efforts, and coalition members reported that having a lead agency was helpful in moving efforts forward and keeping the community engaged.

Through telephone interviews, HPA identified the following lessons learned for other community coalitions:

*Spend time recruiting the “right” members.* Coalition leaders felt they did not have enough time to vet prospective members during early phases of recruitment for sectorial representation, experience and interest in the coalition’s work, and the availability to be involved. Over time, HPA recruited members engaged and able to contribute to HPA’s mission.

*Develop a clear action plan and modify as needed.* A community action plan was useful in organizing and focusing their work. HPA also recommended starting with fewer strategies to attain and build on early success.

*Look for resources beyond staff.* HPA reported the KCHD volunteer’s time, energy, and interest in rural health allowed HPA to accomplish their goals.

*Keep abreast of what is happening in the community.* Identify areas of momentum and interest and look for ways to support those efforts. HPA members attend community events and meetings to stay informed about community needs and activities.

*Be open to new opportunities.* Although the coalition had a well-defined plan, leaders mentioned several instances where a conversation led to new joint efforts. For example, in conversations with a large employer, HPA discovered the owner was an avid bicyclist and secured his commitment to develop land to enhance physical activity opportunities.

*Use any opportunity to promote your work and engage partners.* HPA recommended raising awareness through community events and screening films, such as *Unnatural Causes*, and building on momentum of those events by nurturing relationships with key stakeholders. Community-wide awareness campaigns elevated discussion of connections to health in housing, schools, and other key sectors for community health.

Overall, Klickitat County had a positive experience and increased capacity to implement sustainable approaches proven to reduce risk factors for chronic disease. Their work continues as they pursue new partnerships to donate fresh produce to food banks and achieve policies to support Complete Streets and Safe Routes to School.
